# Validation of echo planar imaging based diffusion-weighted magnetic resonance imaging on a 0.35 T MR-Linac

**DOI:** 10.1016/j.phro.2024.100579

**Published:** 2024-04-20

**Authors:** Philipp Wallimann, Marco Piccirelli, Sylwia Nowakowska, Tess Armstrong, Michael Mayinger, Andreas Boss, Andrea Bink, Matthias Guckenberger, Stephanie Tanadini-Lang, Nicolaus Andratschke, Bertrand Pouymayou

**Affiliations:** a Department of Radiation Oncology, University Hospital Zurich and University of Zurich, Zurich, Switzerland; b Department of Neuroradiology, Clinical Neuroscience Center, University Hospital Zurich and University of Zurich, Zurich, Switzerland; c Institute for Diagnostic and Interventional Radiology, University Hospital Zurich and University of Zurich, Zurich, Switzerland; d ViewRay Inc., 2 Thermo Fisher Way, Oakwood Village, OH 44146, USA

**Keywords:** MR-Linac, quantitative MRI, Diffusion-weighted imaging

## Abstract

**Background and Purpose:**

The feasibility of acquiring diffusion-weighted imaging (DWI) images on an MR-Linac for quantitative response assessment during radiotherapy was explored. DWI data obtained with a Spin Echo Echo Planar Imaging sequence adapted for a 0.35 T MR-Linac were examined and compared with DWI data from a conventional 3 T scanner.

**Materials and Methods:**

Apparent diffusion coefficient (ADC) measurements and a distortion correction technique were investigated using DWI-calibrated phantoms and in the brains of seven volunteers. All DWI utilized two phase-encoding directions for distortion correction and off-resonance field estimation. ADC maps in the brain were analyzed for automatically segmented normal tissues.

**Results:**

Phantom ADC measurements on the MR-Linac were within a 3 % margin of those recorded by the 3 T scanner. The maximum distortion observed in the phantom was 2.0 mm prior to correction and 1.1 mm post-correction on the MR-Linac, compared to 6.0 mm before correction and 3.6 mm after correction at 3 T. In vivo, the average ADC values for gray and white matter exhibited variations of 14 % and 4 %, respectively, for different selections of b-values on the MR-Linac. Distortions in brain images before correction, estimated through the off-resonance field, reached 2.7 mm on the MR-Linac and 12 mm at 3 T.

**Conclusion:**

Accurate ADC measurements are achievable on a 0.35 T MR-Linac, both in phantom and in vivo. The selection of b-values significantly influences ADC values in vivo. DWI on the MR-Linac demonstrated lower distortion levels, with a maximum distortion reduced to 1.1 mm after correction.

## 1 Introduction

The emergence of Magnetic Resonance guided radiotherapy has allowed for improved adaptation of radiotherapy to inter- and intra-fractional motion. This is possible on hybrid Magnetic Resonance (MR) Imaging (MRI) and linear accelerator (Linac) systems, MR-Linacs, which can perform imaging of the patient in the treatment position with good soft tissue contrast and without delivering any additional ionizing radiation.

MRI can provide information beyond the anatomy of soft tissue, for example in the form of angiography, diffusion-weighted imaging (DWI), perfusion-weighted imaging, or spectroscopy. Such advanced imaging at MR-Linacs is under investigation for improved response assessment and new possibilities of treatment adaptation [1], [2], [3].

DWI in particular is of interest as a biomarker in radio-oncology because of its sensitivity to microscopic changes and its inherent quantifiability in terms of apparent diffusion coefficient (ADC) [4], [5], [6], [7], [8], [9]. ADC values are believed to be independent of the used magnetic field strength [10].

DWI has been investigated on a 1.5 T MR-Linac [1], [11], [12], [13], [14], [15], [16] and a 0.35 T MR-Linac [17], [18], [19], [20], [21], [22], [23], [24]. Existing studies involving a 0.35 T MR-Linac have not systematically compared normal tissue ADC values or in-vivo distortion to a clinical scanner. This is particularly relevant to enable a comparison with existing clinical data. Furthermore, there is a need to investigate the impact of b-value selection on the ADC values in the MR-Linac context.

Using DWI as a reliable biomarker for response assessment in radiotherapy requires the ADC values to be reproducible and the geometric distortion in the images to be small. This is particularly challenging as Echo Planar Imaging (EPI) based DWI sequences are highly susceptible to distortion due to inhomogeneities in the magnetic field [25].

We use a spin echo (SE), single shot EPI DWI sequence [25] adapted for the 0.35 T MR-Linac by the vendor. The ADC accuracy and geometric distortion of the sequence was investigated in DWI calibrated phantoms and in brain images of healthy volunteers, compared to a clinical sequence on a conventional 3 T MRI scanner.

## 2 Materials and methods

### 2.1 MRI acquisition and hardware

The DWI sequence investigated on the 0.35 T MRIdian MR-Linac (ViewRay, Denver, CO, USA) [26] was a 2D Spin Echo (SE) single shot Echo Planar Imaging (ssEPI) sequence. The imaging parameters are summarized in Table A.1. The acquisition protocol was chosen according to recommendations of the manufacturer and similar to previous works [17], [21], [22], [24]. Each DWI was acquired with two different phase encoding directions, anterior to posterior and posterior to anterior, with otherwise identical settings.

The voxel size was large, $3\times 3\times 6{\mathrm{mm}}^{3}$, to compensate for the lower signal to noise ratio at the low magnetic field strength. For the volunteers, we used an immobilization device (Civco Medical Instruments, Coralville) that allows for brain imaging with the manufacturer designed 10-channel head & neck coil (ViewRay, Denver, CO, USA) (Figure A.1).

A 3 T clinical MRI scanner (Vida Fit, Siemens Healthineers, Erlangen, Germany) with a similar 2D SE-ssEPI DWI sequence was used for comparison. The acquisition protocols were imported from clinically used brain protocols in order to create a realistic comparison. The b-values and diffusion directions were not aligned with the MR-Linac protocol.

The DWI acquired at the MR-Linac were reconstructed without a prescan normalize filter (Figure A.2) so that the uniform background noise could be used for a noise correction method [27].

All acquired DWI were post-processed with the distortion correction method Topup [28] from the software library FSL [29], which was previously validated for the MR-Linac [21].

For the volunteers, a T1-weighted image was additionally acquired on each scanner to allow tissue segmentation.

### 2.2 Phantoms

**Fig. 1:**
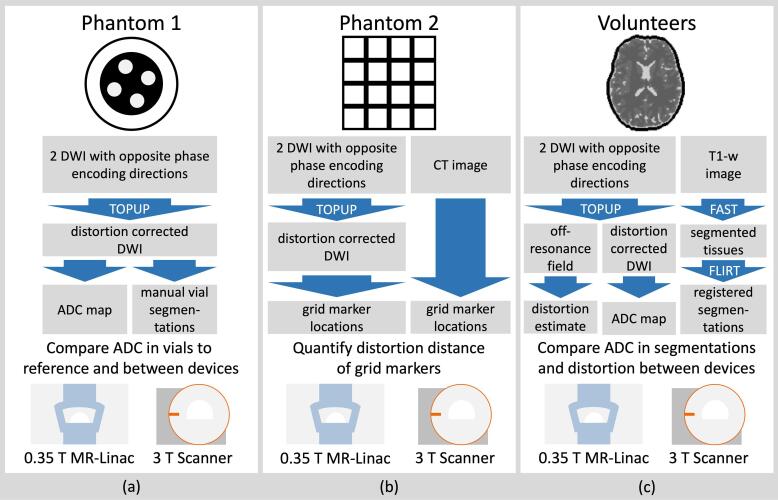
Overview of the conducted DWI evaluation experiments and their corresponding processing workflows at 0.35 T and 3 T. Topup, FAST and FLIRT refer to a distortion correction, segmentation and registration function respectively, as implemented in the FSL software library. (a) Calibrated ADC measurements. (b) Distortion quantifications. (c) Volunteer brain ADC and distortion estimation.

Two phantoms were used in this work. Phantom 1 (HQ imaging, Heidelberg) was used to validate the ADC measurements (Fig. 1a). It contained four vials with calibrated ADC values (at 20 °C) of 400, 1000, 1600 and $2020{\mu m}^{2}/s$ and a built-in thermometer.

Phantom 2 was used to assess the geometric distortion of the DWI. It was created in-house and consisted of a 3D printed grid submerged in water. The grid was made of polylactide, the individual beams had a thickness of 6 mm and were spaced 25 mm apart. The five grid lines along each axis formed a total of $5^{3}$ markers at the intersection points in a field of view of $10\times 10\times 10{\mathrm{cm}}^{3}$. We evaluated the 3D printing accuracy of the grid before it was submerged in water by acquiring a CT scan with resolution $0.78\times 0.78\times 0.6{\mathrm{mm}}^{3}$.

### 2.3 Volunteers

Seven healthy volunteers (4 male, 3 female, mean (±SD) age 29 (±5) years) were recruited. Brain DWI and T1-w images were acquired at the MR-Linac and the 3 T scanner within a time span of a few hours (Fig. 1c). Informed consent was given by each volunteer, all institutional guidelines were followed in the acquisition and handling of the data. The study has been approved by the Local Ethics Committee (BASEC-Nr. 2021-D0066).

The brain mask was extracted from the T1-w images and 3 tissue types were segmented (cerebrospinal fluid (CSF), gray matter (GM) and white matter (WM)). The three tissue segmentations were registered onto the DWI. Details are provided in Supplementary Material A.

### 2.4 Evaluation of apparent diffusion coefficient values

The ADC values were calculated voxelwise using a custom script in Python (Python Software Foundation). For the DWI acquired on the MR-Linac, a correction for a uniform background noise floor was applied [27]. The background noise was determined using a manually selected cuboid volume (Figure A.2). Further details about the calculation and noise correction are provided in Supplementary Material A.

To investigate the impact of the choice of b-values, we calculated two variants of the ADC for the MR-Linac: ${\mathrm{ADC}}_{\mathrm{all}}$, which includes data from all b-values, and ${\mathrm{ADC}}_{0,800}$, which includes only data from the $b=0s/{\mathrm{mm}}^{2}$ and $b=800s/{\mathrm{mm}}^{2}$ images. The ADC at the 3 T scanner, denoted ${\mathrm{ADC}}_{3T}$, was calculated based on the two acquired b-values $b=0s/{\mathrm{mm}}^{2}$ and $b=1000s/{\mathrm{mm}}^{2}$.

The ADC values were evaluated in volumes of interest (VOI). We report the mean and median ADC, as well as the standard deviation and nonparametric skew of the ADC. The standard deviation and skew quantify the heterogeneity within a VOI.

For phantom 1, one VOI per vial was manually selected as a cylindrical volume with radius 6 mm and height 39 mm.

To compare the ADC values calculated within the vials to the reference values, we recorded the temperature value of the built-in thermometer at the time of acquisition. We performed a temperature correction according to the phantom manual (Figure A.3). The relative difference between two measurements in a vial was expressed as follows: $\left({\mathrm{ADC}}_{1}-{\mathrm{ADC}}_{2}\right)/{\mathrm{ADC}}_{\mathrm{reference}}$.

For the volunteers, the three tissue segmentations were used as VOIs. For each VOI, we compared the mean and median ADC per subject between each pair of the three ADC calculation variants using a two-tailed paired Wilcoxon signed-rank test. A p-value of 0.05 was considered as the threshold for significance.

### 2.5 Evaluation of geometric distortion

For phantom 2, the grid markers were detected on the CTs and on the DWI using a template matching approach implemented in MATLAB (MathWorks, Natik). The distortion was defined as the Euclidean distance of the identified markers to the theoretical marker position after a rigid registration of the central 27 markers using the Procrustes method [30].

At the MR-Linac, the slice thickness of the images was reduced to 5 mm to better image the distances between the beams. The DWI at the MR-Linac were repeated multiple times with modifications to individual parameters that relate to distortion, specifically the slice orientation (coronal, axial), the receiver bandwidth (low: 1064 Hz/px, high: 1664 Hz/px, normal: 1352 Hz/px) and the gantry angle (180°, 330°). In each scenario, the average and maximum distortion across all markers was computed for each individual b-value and diffusion direction image before and after the distortion correction. The results for the individual b-values and diffusion directions were averaged together to yield the distortion of the scenario.

For the volunteers, the distortion was evaluated based on the off-resonance field created by Topup. Furthermore, the off-resonance values were converted to ppm with respect to the resonance (14.707 MHz at the MR-Linac and 123.152 MHz at the 3 T scanner), and to a distortion distance in the phase-encoding direction by using the receiver bandwidth in phase-encoding direction as described in Supplementary Material A.

For these quantities, the 95th percentile of the absolute value in the brain is reported as a measure of high, locally occurring values.

## 3 Results

### 3.1 Phantoms

For phantom 1, the temperature at acquisition was determined to be 19.5 °C for both devices.

**Fig. 2:**
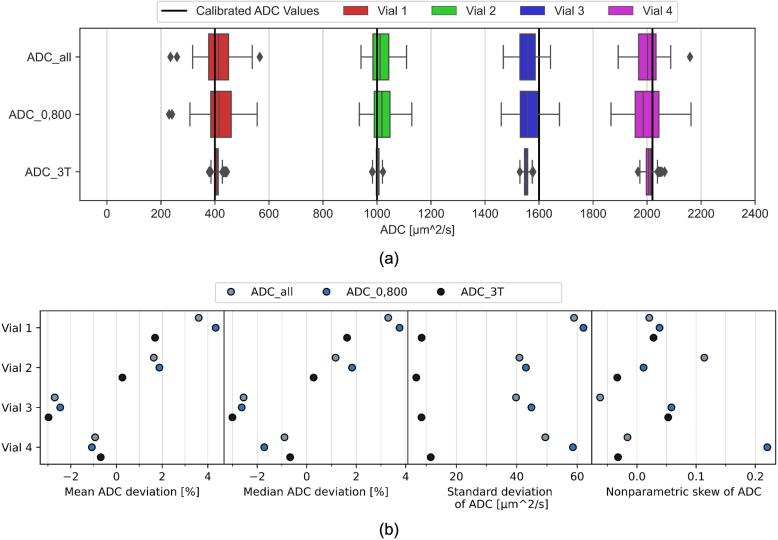
(a) ADC values in each vial of phantom 1 calculated at the MR-Linac using different methods and at the 3 T scanner. (b) Relative difference of the mean and median ADC from the calibrated value, as well as the standard deviation and nonparametric skew of ADC for each vial and calculation method.

The ADC values showed small differences of the mean and median between the MR-Linac and the 3 T scanner at or below 2.7 % (Fig. 2a). Standard deviations of ADC were slightly higher for ${\mathrm{ADC}}_{0,800}$ than for ${\mathrm{ADC}}_{\mathrm{all}}$, and were much lower at ${\mathrm{ADC}}_{3T}$ (Fig. 2b). The nonparametric skews of most ADC distributions in the phantom vials were low with absolute values below 0.1.

The segmented vials and the b-value dependence of the acquired signal intensities are shown in the Supplemental Figure A.4.

**Fig. 3:**
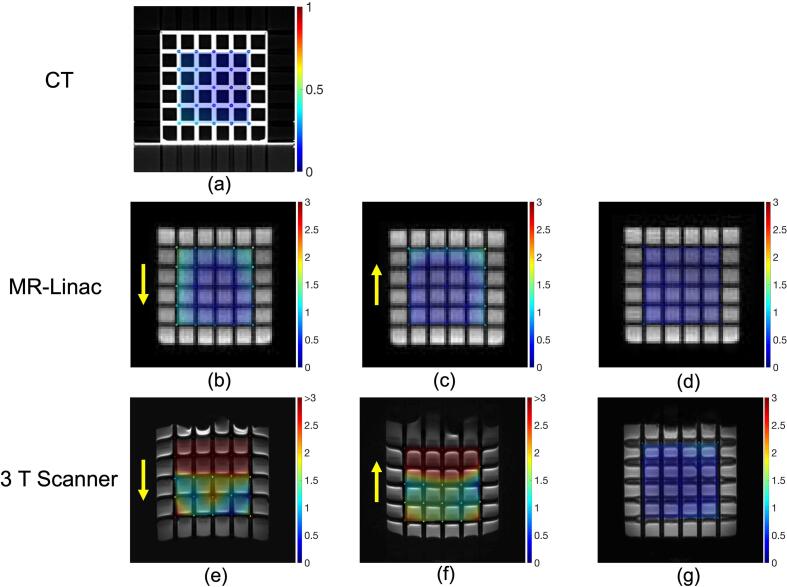
(a) Central axial CT slice of the grid. (b,c, e, f) Central axial slice of DWI with phase encoding direction shown with yellow arrows. (d, g) Central axial slice of DWI after Topup distortion correction. The colored points in all images represent the detected grid location. The color represents the distortion in mm and the scale is provided beside each image. The background color wash visualizes the interpolated distortions in space. (For interpretation of the references to color in this figure legend, the reader is referred to the web version of this article.)

For phantom 2, the mean manufacturing accuracy of the grid locations based on the CT scan (Fig. 3a) was 0.22 ± 0.09 mm (maximum 0.51 mm). A slice of the acquired DWI for both phase encoding directions and the Topup corrected image are shown in Fig. 3b-g for both devices.

For the MR-Linac, the corrected images showed a mean distortion of 0.5 mm, while the maximum distortion varied between 0.96 mm and 1.6 mm (Table 1).

**Table 1:** Mean and maximum distortion of the phantom 2 grid points observed for the different acquisitions. Each value is determined for every single b-value acquisition and then averaged over all acquisitions for a given scenario. The * for the 3 T scanner data indicates that several points of the grid could not be detected by the template matching procedure.

	Acquisition	Default	Coronal orientation	Low BW	High BW	Gantry angle 180°	MR-Linac Average (±SD)	3 T Scanner
Mean distortion [mm]	Image	0.9	0.8	1.0	0.8	1.0	0.87 ± 0.10	3.5*
	Reverse phase encoding image	0.7	0.8	0.8	0.7	0.8	0.78 ± 0.06	3.6*
	Topup corrected image	0.5	0.5	0.4	0.5	0.5	0.49 ± 0.03	0.8
Maximum distortion [mm]	Image	2.0	1.8	2.2	2.3	2.3	2.11 ± 0.23	5.8*
	Reverse phase encoding image	1.6	1.9	1.9	2.2	2.2	1.95 ± 0.22	6.0*
	Topup corrected image	1.1	1.6	1.0	1.2	1.2	1.22 ± 0.24	3.6

For the 3 T scanner, the mean distortion was 0.76 mm after correction, but visual inspection of the image shows blurriness for the horizontal grid lines (Fig. 3g). Some grid markers could not be detected in the uncorrected images, meaning the uncorrected distortions reported at 3 T are underestimations.

### 3.2 Volunteers

**Fig. 4:**
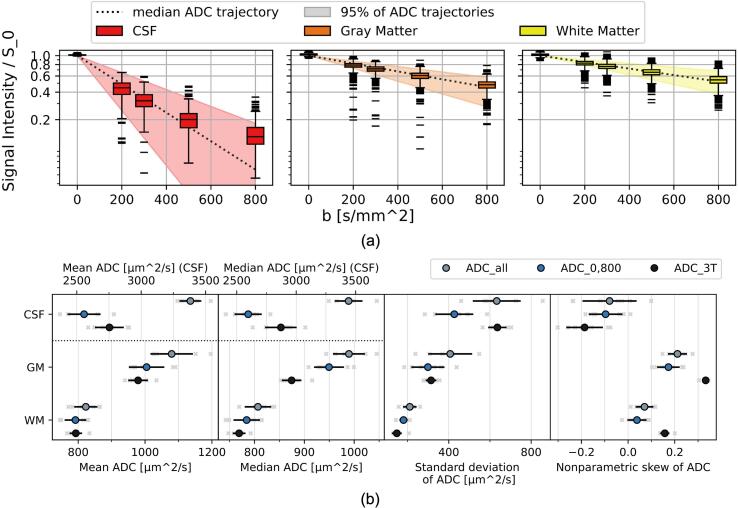
(a) Box plots of signal intensity values after noise correction divided by the baseline signal S0 obtained from the fit for one subject at the MR-Linac for the tissues and b-values. Median ADC value trajectories and trajectory intervals containing 95% of ADC values are shown. (b) Mean, median, standard deviation and nonparametric skew of ADC for each subject, tissue and calculation method. Values obtained from individual subjects shown as light gray crosses, population means as colored points and population standard deviations as black lines.

For the volunteers, the signal intensities do not show a clear mono-exponential dependence on the b-values, in particular in CSF (Fig. 4a). We observed a larger spread of intensities and ADC than in the phantom.

For each VOI, the mean and median ADC values per subject (Fig. 4b) were compared between calculation methods using a two-tailed paired Wilcoxon signed-rank test and the resulting p-values are visible in Table A.2. All comparisons showed a significant difference except for those between ${\mathrm{ADC}}_{0,800}$ and ${\mathrm{ADC}}_{3T}$ in GM for the mean ADC and in WM for both the mean and median ADC.

The standard deviations in CSF and GM were much larger for ${\mathrm{ADC}}_{\mathrm{all}}$ than the other approaches. For ${\mathrm{ADC}}_{3T}$, the standard deviation in CSF was also larger than for ${\mathrm{ADC}}_{0,800}$. In contrast to the phantom 1 results, all ${\mathrm{ADC}}_{3T}$ distributions in vivo were strongly skewed. The results in WM showed the lowest skew.

Supplementary Material A provides further visualizations of the data (Figure A.5) and of the noise correction (Figure A.6). Due to user error, subjects 2 and 6 were not reconstructed without the prescan normalize filter, leading to a slightly underestimated noise magnitude for those two cases.

**Fig. 5:**
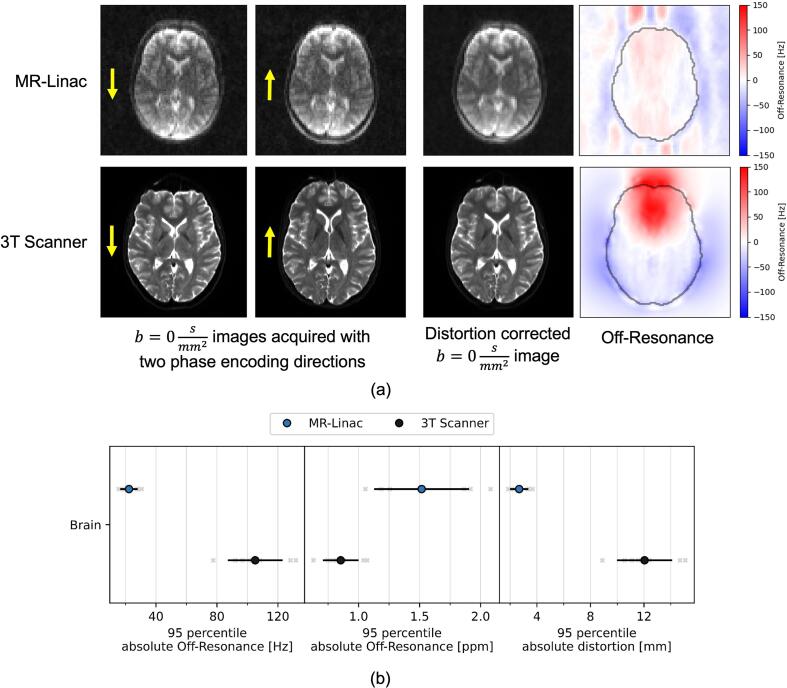
(a) A slice of the $b=0s/{\mathrm{mm}}^{2}$ images acquired for a subject at both scanners in two phase encoding directions each, as indicated by the yellow arrows. The distortion-corrected image and the estimated off-resonance field, as created by Topup. The brain contour is shown in black. (b) 95th percentile values of the absolute off resonance and distortion values in the brain for each subject and device. Values obtained from individual subjects shown as light gray crosses, population means as colored points and population standard deviations as black lines. (For interpretation of the references to color in this figure legend, the reader is referred to the web version of this article.)

The distortions of the uncorrected $b=0s/{\mathrm{mm}}^{2}$ images were larger at the 3 T scanner, which also corresponded to a higher off-resonance estimated by Topup (Fig. 5a).

In terms of 95th percentile values, the MR-Linac showed lower absolute off-resonance and calculated distortion, but higher ppm off-resonance (Fig. 5b).

## 4 Discussion

We intended to assess the performance of diffusion-weighted imaging on a 0.35 T MR-Linac compared to a 3 T clinical scanner. We investigated the geometric distortion and accuracy of apparent diffusion coefficient values in phantoms and in vivo.

The phantom 1 experiment demonstrated a high consistency between the ADC measurements on the two machines, with mean and median ADC values deviating by 2.7 % or less. However, the resolution of ADC maps and the precision of ADC values were lower at the MR-Linac. The acquisition time was substantially longer at the MR-Linac, but because the patient is already in treatment position, the longer time spent for the image may be acceptable. These results are similar to the ones reported previously [17], [19], [21], [23], [24] but do not show the underestimation of ADC reported by Lewis, et al. [22]. Both scanners showed a similar underestimation of ADC in vial 3, indicating that the vial might have deviated from the calibrated value.

The relationship between signal intensities and b-values was consistent with the mono-exponential ADC model and the ${\mathrm{ADC}}_{0,800}$ fit produced only minor (<1%) differences of the mean and median ADC compared to ${\mathrm{ADC}}_{\mathrm{all}}$. ADC distributions in the phantom vials showed low skew, with the exception of vial 4 for ${\mathrm{ADC}}_{0,800}$.

The phantom 2 experiment demonstrates the efficacy of the Topup distortion correction for both scanners. The maximum distortion observed at the MR-Linac was reduced to 1.1 mm (8.6 cm from the isocenter). This is close to the standard for planning images at the MR-Linac of distortion below 1 mm in a radius of 10 cm, which was also reported for clinical images at the 0.35 T MR-Linac by Hasler, et al. [31]. Before the correction, maximum distortions over 2 mm were observed, which is consistent with the 95th percentile of estimated distortion in the volunteers. Images acquired at gantry angle 180° showed higher distortions. As expected, distortions before correction were larger for lower receiver bandwidths. However, after the correction, we observed the opposite effect. It is possible that the improved SNR of the lower bandwidth images was more favorable for the Topup algorithm.

At 3 T, uncorrected phantom 2 images showed large distortions (>5 mm and some points where the template matching failed). This is compatible with the 12 mm estimated by the off-resonance field in the volunteers. The distortion is larger than the one reported by Hasler, et al. [31] for SE-EPI DWI on a 3 T radiology scanner (3.65 mm within 10 cm), likely because they used a higher receiver bandwidth (2779Hz/px). The grid pattern in phantom 2 could not be fully recovered at 3 T, even after the correction, which undermines the good performance of the correction (average distortion 0.76 mm).

The lower distortion expected for a lower magnetic field strength could be confirmed at the MR-Linac. The application of a distortion correction may still be warranted if very high geometric accuracy is necessary. For clinical scanners, more advanced DWI sequences may be available to mitigate the distortions [32], [33].

The volunteer experiment allows for a comparison of normal brain tissue ADC values between the 0.35 T MR-Linac and a clinical scanner. The parameters related to diffusion encoding (e.g. b-values or diffusion time [34], [35]) were not strictly harmonized between the scanners. That represents a realistic situation, but has to be considered when interpreting the results. Inaccuracies in the segmentation also have to be considered as the DWI had a lower resolution and may still exhibit residual distortion after correction.

The ADC distributions in CSF and GM were more skewed than in the phantom experiment. For the 3 T scanner, high skew was observed in all VOIs. This implies that ADC values are heterogeneous even within an acquisition and that the mean or median ADC values may provide an incomplete picture. A reason for this may be that voxels in the VOIs contain undesired tissue due to the partial volume effect (PVE). For the 3 T scanner, the fraction of PVE voxels is assumed to be low due to the high resolution, which is consistent with the observed behavior of clear peaks in the ADC distribution but large tails with deviating values. For the MR-Linac, the lower resolution means PVE potentially affects a larger fraction of voxels, leading to an overall shift in the distribution.

The mean and median ADC differed significantly between ${\mathrm{ADC}}_{\mathrm{all}}$ and ${\mathrm{ADC}}_{0.800}$ in each VOI. This indicates that the signal intensity does not show a mono-exponential dependence on the b-values, for example due to perfusion effects [36]. For CSF we found unrealistically high ${\mathrm{ADC}}_{\mathrm{all}}$ values, which may be a result of bulk motion [36]. The b-value dependence was not present in phantom 1, which highlights the need for further b-value alignment in the context of a prospective clinical data acquisition.

For CSF, mean and median ADC for ${\mathrm{ADC}}_{0.800}$ were lower than for ${\mathrm{ADC}}_{3T}$, which may be due PVE or differences in the imaging parameters, such as the TR [5] or b-values [35]. This deviation may have little clinical relevance.

For GM, the mean ADC values showed no significant difference between ${\mathrm{ADC}}_{0.800}$ and ${\mathrm{ADC}}_{3T}$ while the median did. The substantial difference in the medians is larger than would be expected from differences in diffusion times [34], but may be caused in part by the different maximum b-values [35]. Segmentation uncertainty is also a likely cause due to the low resolution at the MR-Linac.

For WM, both the mean and median ADC showed no significant difference between ${\mathrm{ADC}}_{0.800}$ and ${\mathrm{ADC}}_{3T}$. It was the VOI least affected by PVE. It is possible that the discrepancy between the scanners in other VOIs could also be reduced with a more harmonized acquisition and a higher resolution at the MR-Linac.

The off-resonance values estimated in the brain align with expectations, e.g. a positive value above the sinuses [37] and a higher off-resonance at the stronger magnetic field. However, relative off-resonance in terms of ppm was lower for the 3 T scanner, possibly due to improved shimming on that system.

Ultimately, the 0.35 T MR-Linac can acquire ssEPI DWI with lower distortions compared to a 3 T scanner. The ADC values were not significantly different between the scanners for the phantom vials and healthy WM. It is possible that this finding can be extended to other tissues and sites as long as the b-values between the machines are chosen close enough and the volume of interest can be segmented well. Using 5 b-values in vivo did not produce reliable results for a mono-exponential model, but may be explored for example with an IVIM model in the future [36], [38]. Using two b-values $0\;s/{\mathrm{mm}}^{2}$ and $800\;s/{\mathrm{mm}}^{2}$ for brain DWI at the 0.35 T MR-Linac results in ADC values that can be compared to similarly acquired images at clinical scanners in homogeneous tissue.

## CRediT authorship contribution statement

**Philipp Wallimann:** Conceptualization, Software, Investigation, Writing – original draft, Visualization. **Marco Piccirelli:** Investigation, Writing – review & editing. **Sylwia Nowakowska:** Investigation, Writing – review & editing. **Tess Armstrong:** Software, Writing – review & editing. **Michael Mayinger:** Resources, Writing – review & editing. **Andreas Boss:** Conceptualization, Writing – review & editing, Funding acquisition. **Andrea Bink:** Resources, Writing – review & editing. **Matthias Guckenberger:** Resources, Writing – review & editing. **Stephanie Tanadini-Lang:** Conceptualization, Supervision, Resources, Writing – review & editing, Funding acquisition. **Nicolaus Andratschke:** Conceptualization, Supervision, Resources, Writing – review & editing, Funding acquisition. **Bertrand Pouymayou:** Conceptualization, Supervision, Investigation, Software, Resources, Writing – review & editing, Visualization.

## Declaration of competing interest

The authors declare the following financial interests/personal relationships which may be considered as potential competing interests: PW, MM, NA, BP have previously received compensation from ViewRay, Inc. for consulting work. For a time period during this project, TA was employed at ViewRay, Inc. and owned stocks in ViewRay, Inc.
