# Supplementary material for: Validation of echo planar imaging based diffusion-weighted magnetic resonance imaging on a 0.35 T MR-Linac
Source: Phys Imaging Radiat Oncol. 2024 Apr 20;30:100579. doi: 10.1016/j.phro.2024.100579 (PMC11068927; doi:10.1016/j.phro.2024.100579)
Supplement: Supplementary Data 1 [file mmc1.pdf]

Supplementary Material A.

Brain Image Segmentation and Registration

The brain volume was extracted from the T1-w images using HD-BET [1] with default parameters. The T1-w images were automatically segmented within the brain masks into the three tissue types cerebrospinal fluid (CSF), gray matter (GM) and white matter (WM) using the FAST function [2] of the FSL software library [3]. For each tissue type, a partial volume effect (PVE) image was created by FAST, which contains for each voxel the confidence of the classification to the given tissue on a scale from 0 to 1. The T1-w images were linearly registered and resampled to the distortion-corrected DWI coordinates (to the $b = 0\ s/mm^2$ image) using the function FLIRT [4,5]. The same transformation was applied to the brain volume and the PVE segmentation images in order to transform them into the DWI coordinates.
After the registration, a threshold of 0.8 was applied to the PVE images in order to create binary masks. For the CSF, we restricted the analysis to the homogeneous region of the ventricles by excluding voxels that were not in the brain mask after a 20 mm erosion thereof.
The resulting segmentations on the T1-w image and ADC map for one slice of one subject is shown in figure A.5(a).

DWI Postprocessing

The susceptibility distortion in each DWI was corrected using the Topup function [6] from the software library FSL [3]. The function uses image data from the two $b = 0\ s/mm^2$ acquisitions with opposite phase encoding directions to estimate an off-resonance field. The off-resonance field is used to apply a susceptibility distortion correction to the pair of images with opposite phase encoding direction at each b value and gradient direction. The resulting images are substantially more spatially accurate, although distortions due to eddy currents induced by the diffusion gradients are not compensated in this manner. The corrected images were used for further analysis.

The apparent diffusion coefficient (ADC) was calculated for the MR-Linac data using a voxelwise mono-exponential least-squares fit over the signal intensities of the different b-images. The relationship is expressed with the formula $S_b = S_0\ exp(-b\ ADC)$, where S_b is the signal intensity at the given voxel for a given b value, S_0 is the theoretical baseline intensity of that voxel. Both S_0 and ADC were used as variables in the fit. The data points were all weighted equally for the fit.
Since images with three different diffusion directions were acquired at the MR-Linac, the data of these acquisitions had to be combined in order to calculate a single, isotropic ADC. This was achieved by calculating for each voxel the geometric mean of the signal intensity across the different diffusion gradient directions at each b value before the ADC fit. At the 3 T scanner, we used the trace-weighted images for the higher b-value, which already combines all diffusion directions.
In vivo, ADC values above $4500\ \mu m^2/s$ (about 1.5 times the ADC of free water at 37°) were considered as measurement errors and thus ignored. (For ADC_{all}, this was the case for about 21% of CSF voxels, 1% of GM voxels and less than 0.01% of WM voxels. For $ADC_{0,800}$ and ADC_{3T}, a failure of fit was the case for less than 0.01% of voxels in all tissues)
For DWI at low SNR, the non-zero noise floor that exists in the images can lead to biased intensity values, particularly at images with high b values [7]. A correction of the intensities for uniform Rician noise was applied prior to the ADC fit at the MR-Linac with the following formula:

$$S_{corr} = \sqrt{S^2 - (2/\pi)N^2},$$

where S_{corr} is the corrected intensity value at a given voxel, S is the acquired intensity value at the voxel and N is the mean intensity in the background of the image [7]. Inhomogeneities in the noise distribution introduced by parallel imaging were neglected in this case. We determined a background mask in the images as a large, manually selected cuboid volume, as shown in Figure A.2.

To quantify the distortion of the images, the off-resonance field estimated by Topup was used to calculate the magnitude of distortion along the phase encoding direction at each point in the image using the formula

$$\Delta x = (\Delta\omega / pBW_p) l_x,$$

where x is the spatial distortion in the phase encoding direction, $\Delta\omega$ is the off-resonance frequency, l_x is the length of a voxel in the phase encoding direction, and pBW_p is the receiver bandwidth per pixel in the phase encoding direction. The latter can be calculated for the ssEPI sequences as
$pBW_p = P/(n_p\, ES)$,
where P is the parallel imaging acceleration factor, n_p is the number of phase encoding steps acquired (not accounting for partial fourier) and ES is the echo spacing. For the sequence at the MR-Linac, we had $P = 2$, $n_p = 100$ and $ES = 0.8\ ms$, therefore $pBW_p = 25\ Hz/px$. For the sequence at the 3 T scanner, we had $P = 2$, $n_p = 192$ and $ES = 1.04\ ms$, therefore $pBW_p = 10\ Hz/px$.

The ADC values obtained without Topup correction, shown in Figure A.7, showed similar accuracy of the median for the 3 T scanner. For the MR-Linac, the values for vials 1 and 2 were higher or lower without Topup correction, depending on the phase-encoding direction. The standard deviation of ADC values was always higher without Topup correction.

Table A.1: Imaging parameters of the MRI sequences used on both devices.

	0.35 T MR-Linac		3 T Scanner	
Sequence	SE ssEPI DWI	3D GRE T1-w	SE ssEPI DWI	3D GRE T1-w
Orientation	Axial	Sagittal	Axial	Sagittal
Flip angle [°]	90	30	90	10
TR [ms]	3200	20	5800	1900
TE [ms]	120	6.23	96	2.17
TI [ms]	-	-	-	957
Receiver bandwidth [Hz/px]	1352	90	1042	220
Voxel size (acquisition) [mm^3]	3×3×6	1.5×1.5×1.5	1.15×1.15×4	0.83×0.83×1
Voxel size (reconstruction) [mm^3]	3×3×6	1.5×1.5×1.5	0.57×0.57×4	0.42×0.42×1
Slice gap [mm]	0	0	1.2	0
Field of view [mm^3]	300×300×120	270×280×216	220×220×134	240×240×192
Number of signal averages	6	1	2 (b=0), 3 (b=1000)	1
Partial Fourier Factor	6/8 (phase)	7/8 (phase), 7/8 (slice)	6/8 (phase)	-
GRAPPA acceleration factor / Reference lines	2 / 24	-	2 / 46	3 / 24

Phase encoding direction	PA & AP	AP	PA & AP	AP
b-values $[s/mm^2]$	0, 200, 300, 500, 800	-	0, 1000	-
Diffusion direction scheme	3 orthogonal directions (read, phase, slice)	-	4 tetrahedral directions (“4 scan trace”)	-
Diffusion gradient scheme	Bipolar	-	Bipolar	-
Fat suppression	-	-	Fat saturation: “Strong”	-
Distortion correction at the scanner	2D	3D	2D	3D
Receiver coil	10 channel head & neck	10 channel head & neck	64 channel head & neck	64 channel head & neck
Coil combination	Adaptive Combine	Adaptive Combine	Adaptive Combine	Adaptive Combine
Gantry angle	330°	330°	-	-
Total acquisition time $[min{:}s]$	8:38	5:52	3:20	3:51

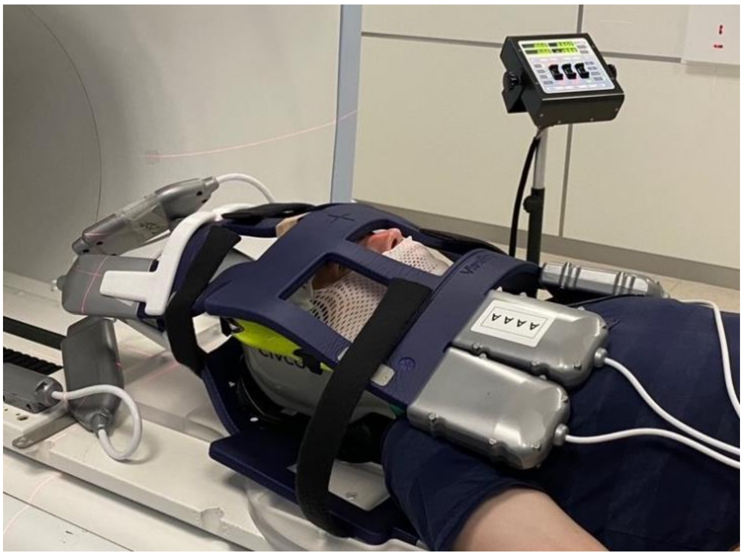

Figure A.1: Head & neck coil setup with immobilization device for brain imaging at the MR-Linac.

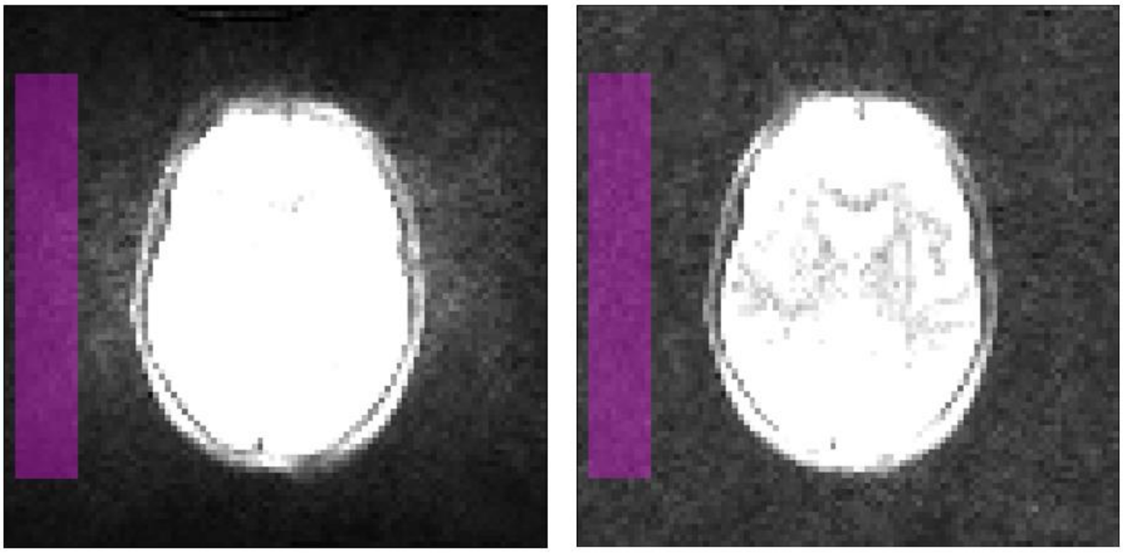

(a) (b)

Figure A.2: (a) A slice of the $b = 0\ s/mm^2$ acquisition for one volunteer with the prescan normalize filter applied. (b) The same slice without prescan normalize filter. In both cases, the purple region represents the manually selected background region. The images are shown with the same intensity window to highlight differences in the background noise pattern.

Diffusion Phantom – Custom Made

ADC Compartments

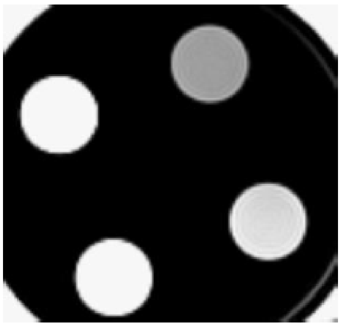

ADC values at 20 °C: 0.4, 1.0, 1.6 and 2.02 µm²/ms.

The ADC of the surrounding solution is 1.2 µm²/ms.

The ADC at 20 °C can be calculated with the following equation:

$$ADC_{20°C} = ADC_{measured} / \exp\left(c \times (T_{measured} - 20°C)\right)$$

whereas

c = 0.0323 1/°C for ADC(20 °C) ~ 0.6 µm²/ms
c = 0.0286 1/°C for ADC(20 °C) ~ 1.0 µm²/ms
c = 0.0278 1/°C for ADC(20 °C) ~ 1.2 µm²/ms
c = 0.0261 1/°C for ADC(20 °C) ~ 1.6 µm²/ms
c = 0.0244 1/°C for ADC(20 °C) ~ 2.02 µm²/ms

Figure A.3: Temperature correction scheme for ADC values described in the manual of phantom 1.

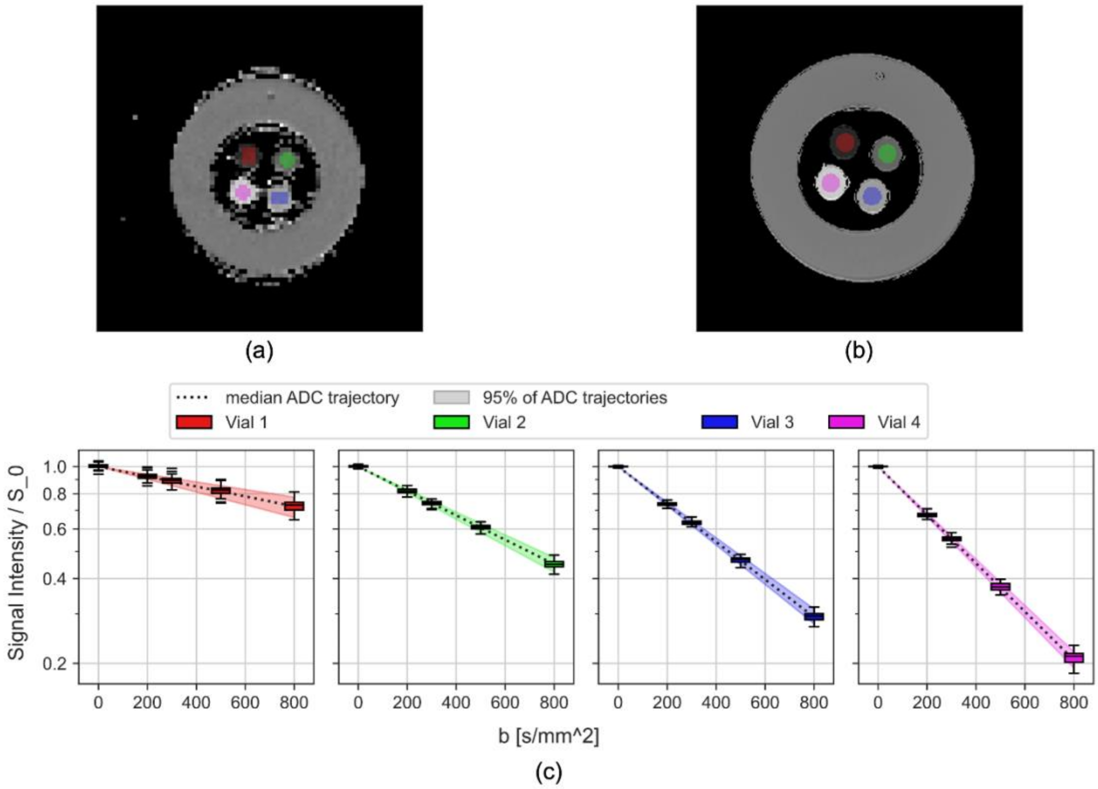

Figure A.4: (a) A slice of the ADC map of the ADC phantom acquired with the MR-Linac along with four contoured vials. (b) The same contours shown on the ADC map acquired with the 3 T scanner. (c) Box plots of signal intensity values after noise correction divided by the baseline signal S0 (as obtained from the fit) at the MR-Linac for the vials and b-values. Median ADC value trajectories and trajectory intervals containing 95% of ADC values are shown.

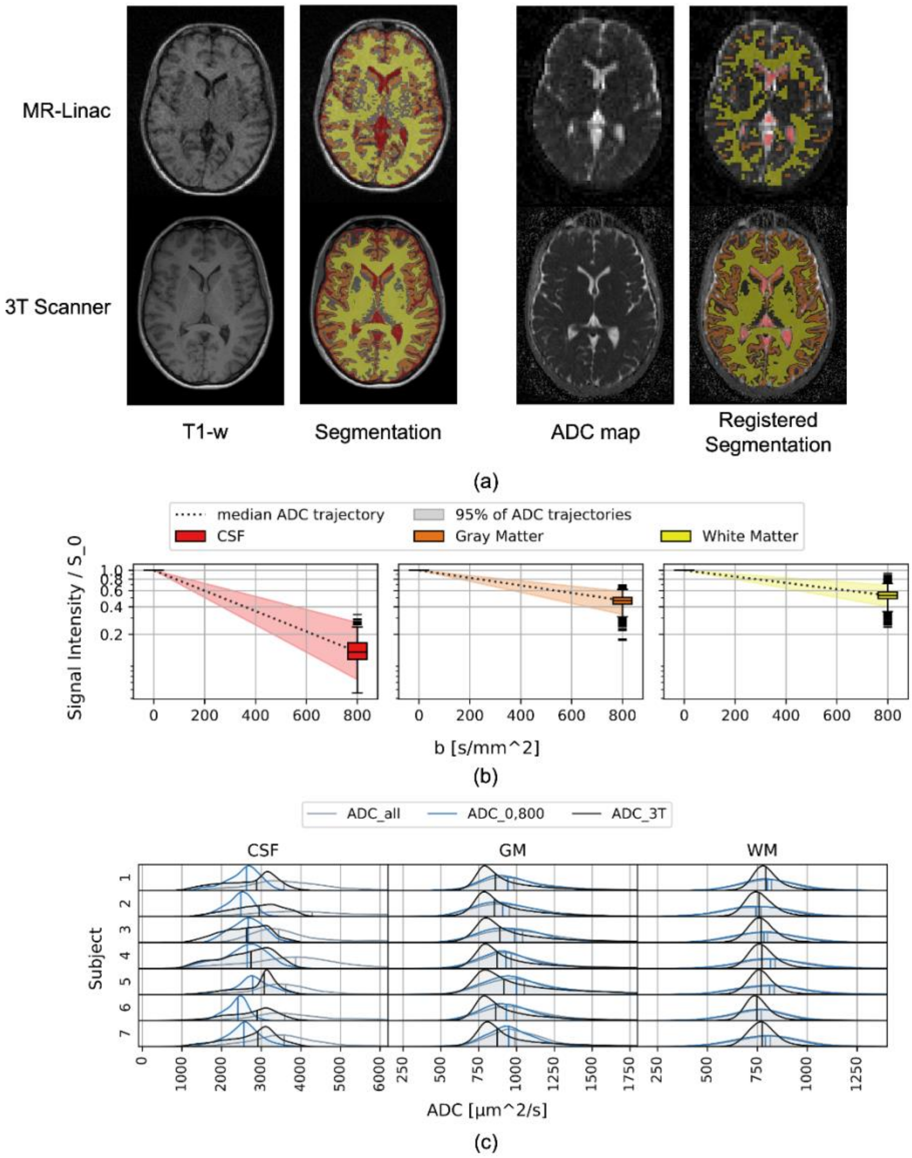

Figure A.5: (a) T1-w scans, ADC maps and segmentations for both scanners for one subject. (b) Box plots of signal intensity values after noise correction divided by the baseline signal S0 obtained from the fit for one subject at the MR-Linac for the tissues and only the two b-values $0\ s/mm^2$ and $800\ s/mm^2$. Median ADC value trajectories and trajectory intervals containing 95% of ADC values are shown. (c) ADC values for each subject and in each tissue calculated at the MR-Linac using different methods and at the 3 T scanner. Kernel density estimation was created with silverman bandwidth estimation. Median ADC values shown as vertical lines in each

case.

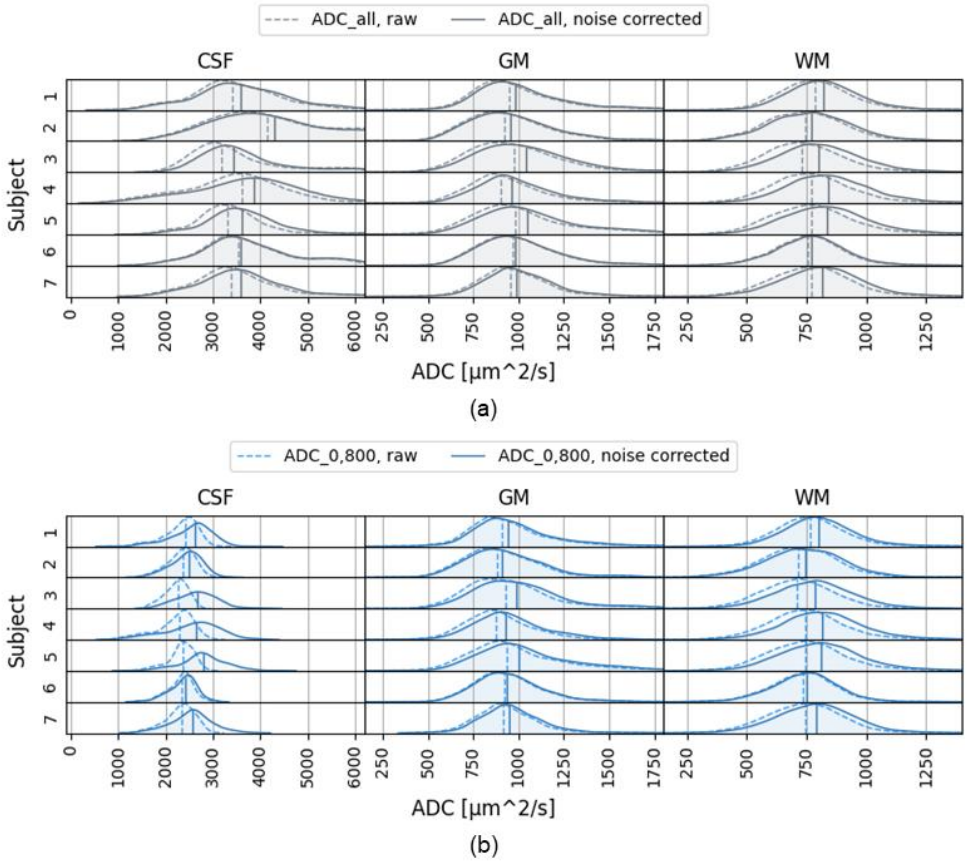

Figure A.6: (a) Histograms comparing the ADC_{all} values for each volunteer subject and in each tissue calculated at the MR-Linac with and without noise correction. Kernel density estimation was created with silverman bandwidth estimation. The vertical lines represent the median ADC value in each case. Subjects 2 and 6 were not reconstructed without the prescan normalize filter, therefore the background noise is underestimated, leading to a smaller noise correction. (b) The same histograms, but for $ADC_{0,800}$.

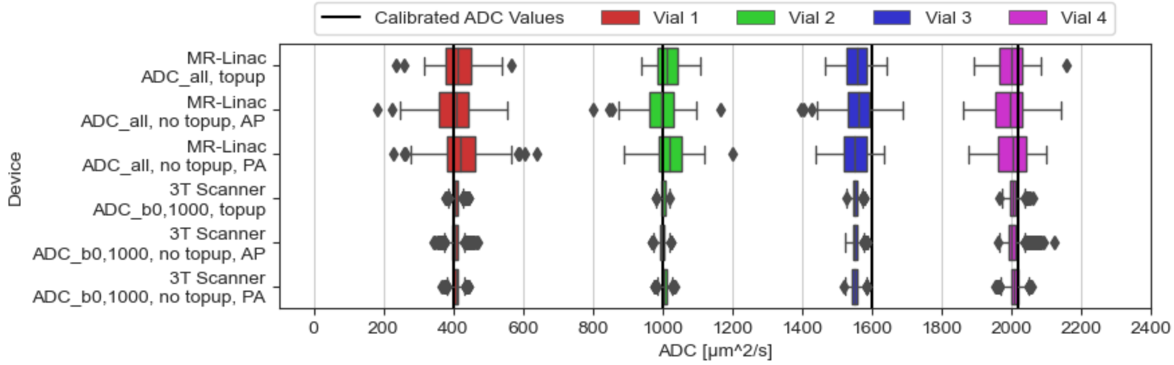

Figure A.7: ADC values determined in phantom vials using distortion corrected image (topup) compared to using only one phase-encoding direction, AP or PA, without distortion correction (no topup). The median at the 3 T scanner deviates by less than 1% between the approaches, but the standard deviations are slightly larger without Topup. For the MR-Linac, the medians for vials 3 and 4 also differ by less than 1% between the methods, but for vials 1 and 2, the median is about 1.5% lower or higher without Topup, depending on the phase encoding direction. The standard deviations are also slightly larger without Topup.

Table A.2: The p-values of the two-tailed paired Wilcoxon signed-rank tests between the different ADC measurements to compare the mean and median ADC values within each VOI for all subjects. Repeated p-values are a result of the same outcome of the test statistic.

p-values of comparison between ADC calculation methods	Tissue	Mean ADC in segmentation	Median ADC in segmentation
ADC_{all} compared to $ADC_{0,800}$	CSF	**0.016**	**0.016**
	GM	**0.016**	**0.016**
	WM	**0.016**	**0.016**
ADC_{all} compared to ADC_{3T}	CSF	**0.016**	**0.016**
	GM	**0.016**	**0.016**
	WM	**0.016**	**0.016**
$ADC_{0,800}$ compared to ADC_{3T}	CSF	**0.031**	**0.031**
	GM	0.156	**0.016**
	WM	0.938	0.156

References

[1] Isensee F, Schell M, Pflueger I, Brugnara G, Bonekamp D, Neuberger U, et al. Automated brain extraction of multisequence MRI using artificial neural networks. Human Brain Mapping 2019;40(17):4952–64. https://doi.org/10.1002/hbm.24750.

[2] Zhang Y, Brady M, Smith S. Segmentation of brain MR images through a hidden Markov random field model and the expectation-maximization algorithm. IEEE Trans Med Imaging 2001;20(1):45–57. https://doi.org/10.1109/42.906424.

[3] Smith SM, Jenkinson M, Woolrich MW, Beckmann CF, Behrens TEJ, Johansen-Berg H, et al. Advances in functional and structural MR image analysis and implementation as FSL. NeuroImage 2004;23:S208–19. https://doi.org/10.1016/j.neuroimage.2004.07.051.

[4] Jenkinson M, Smith S. A global optimisation method for robust affine registration of brain images. Medical Image Analysis 2001;5(2):143–56. https://doi.org/10.1016/S1361-8415(01)00036-6.

[5] Jenkinson M, Bannister P, Brady M, Smith S. Improved Optimization for the Robust and Accurate Linear Registration and Motion Correction of Brain Images. NeuroImage 2002;17(2):825–41. https://doi.org/10.1006/nimg.2002.1132.

[6] Andersson JLR, Skare S, Ashburner J. How to correct susceptibility distortions in spin-echo echo-planar images: application to diffusion tensor imaging. NeuroImage 2003;20(2):870–88. https://doi.org/10.1016/S1053-8119(03)00336-7.

[7] Dietrich O, Heiland S, Sartor K. Noise correction for the exact determination of apparent diffusion coefficients at low SNR. Magn Reson Med 2001;45(3):448–53. https://doi.org/10.1002/1522-2594(200103)45:3<448::AID-MRM1059>3.0.CO;2-W.
